# Behavioral responses of *Drosophila suzukii* to blends of its attractants

**DOI:** 10.17912/micropub.biology.001555

**Published:** 2025-04-22

**Authors:** Kazi Hasan, Hany Dweck

**Affiliations:** 1 Entomology, Connecticut Agricultural Experiment Station, New Haven, Connecticut, United States

## Abstract

*Drosophila suzukii* poses a significant threat to soft-skinned fruits worldwide. Effective trapping of this pest largely depends on commercially available lures, which often capture not only *D. suzukii* but also other species. Previously, we identified phenylacetaldehyde, spermidine, and pyridine as specific attractants for *D. suzukii* . Here we tested mixtures of these odorants and found that a blend of all three odorants did not produce any attraction. However, mixtures of phenylacetaldehyde with either spermidine or pyridine, but not spermidine with pyridine, triggered significant attraction. These findings can guide the formulation of more effective lures for *D. suzukii* .

**Figure 1. Behavioral responses of D. suzukii to blends of its attractants:**
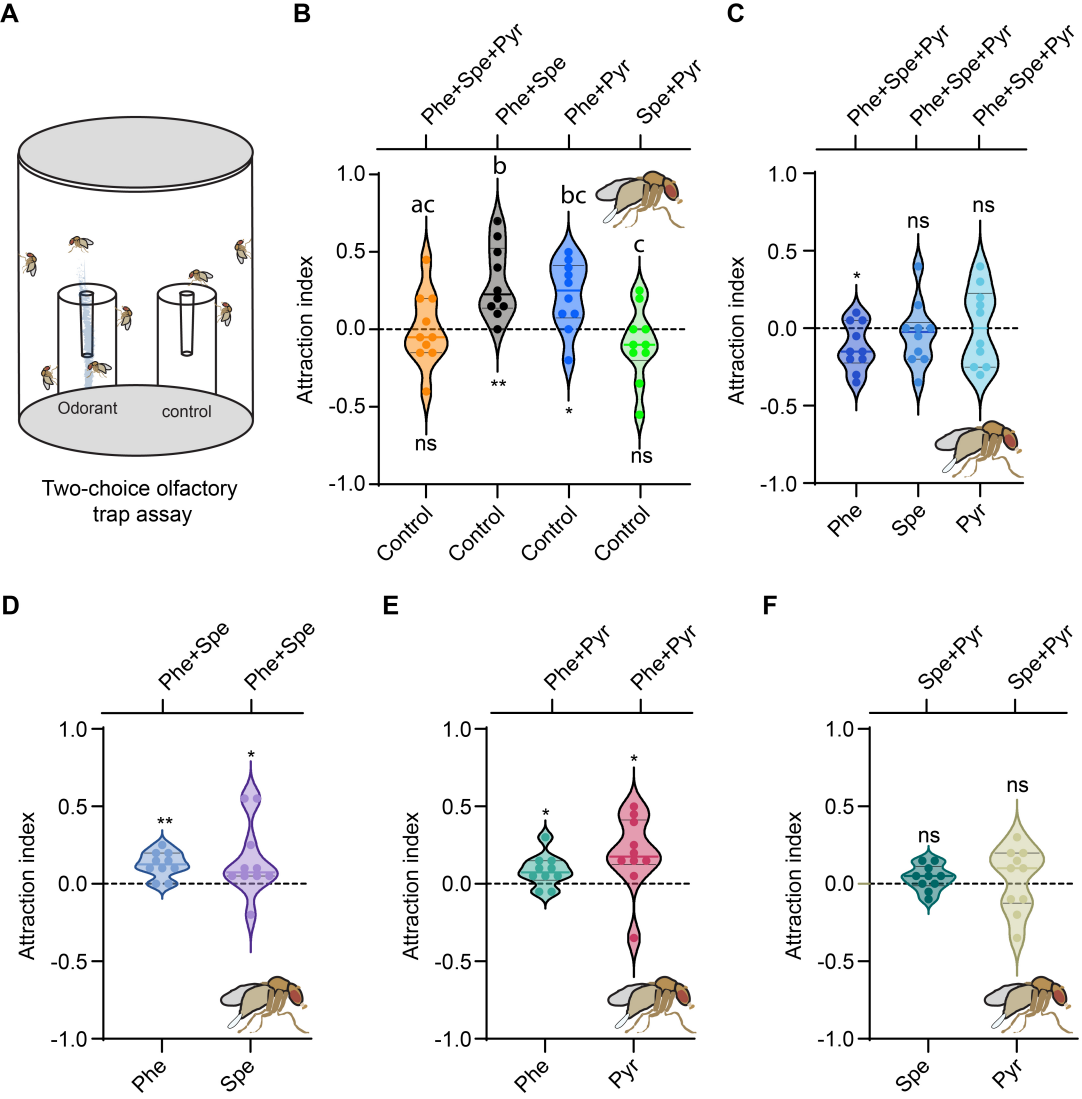
(A) Schematic of the trap assay. (B) Attraction to blends of attractive odorants *.* Data are plotted as violin plots, showing the median, first and third quartiles, and all data points. Each preference index was compared to 0 using the Wilcoxon signed-rank test (*p<0.05, **p<0.01, n = 10). The One-way ANOVA test followed by Tukey’s multiple comparison test was used to compare the preference indices to the different blends. Values indicated with different letters are significantly different (p<0.05). (C-F) Attraction to a blend of phenylacetaldehyde, spermidine, and pyridine (C), a blend of phenylacetaldehyde and spermidine (D), a blend of phenylacetaldehyde and pyridine (E), and a blend of spermidine and pyridine (F). Data are plotted as violin plots, showing the median, first and third quartiles, and all data points. Wilcoxon signed-rank test, n=10.

## Description

The invasive pest *Drosophila suzukii* (spotted wing Drosophila) poses a significant economic threat to fruit industries worldwide (Calabria and others 2012; Deprá and others 2014; Walsh and others 2011). *D. suzukii* females have a heavily sclerotized, serrated ovipositor that allows them to lay eggs in underripe and ripe fruit, leading to larval infestation and microbial infections that severely damage crops (Cavey and others 2023; Dweck and others 2021; Hauser 2011; Karageorgi and others 2017; Lee and others 2011). Additionally, even the detection of a single *D. suzukii* larva can result in the rejection of fruit shipments. These challenges are further intensified by *D. suzukii* ’s high reproductive rate, robust dispersal abilities, and behavioral, physiological, and developmental flexibility (Little and others 2020; Tait and others 2021). These factors can hinder effective monitoring and management efforts and can contribute to a rapid evolution of resistance to conventional insecticides. This situation indicates an urgent need for alternative tools and strategies that can be integrated into *D. suzukii* management programs.

Previously, we identified phenylacetaldehyde (Phe), spermidine (Spe), and pyridine (Pyr) as specific attractants for *D. suzukii* (Xue and others 2025). In this study, we tested the behavioral responses of mated females to blends of these odorants.

We first tested a blend of the three odorants using a two-choice olfactory trap assay ( Figure 1A ). For this experiment, we prepared two traps: one trap contained a mixture of phenylacetaldehyde, spermidine, and pyridine, each at a 0.03% concentration, dissolved in a solution of 0.5% agar and 2% sucrose, while the second trap contained only 0.5% agar and 2% sucrose. Based on our previous findings, we chose to test these odorants at 0.03% concentration, as each odorant was individually attractive to mated females at this concentration (Xue and others 2025).

Surprisingly, when mated females were given a choice between these two traps, they showed no preference for either ( Figure 1B ). This result suggests that the blend of the three odorants did not enhance attraction as expected. This observation led us to hypothesize that the inclusion of one odorant might have negatively impacted the attractiveness of the blend. Therefore, we next tested blends of two odorants.

As hypothesized, mixtures of phenylacetaldehyde with either spermidine or pyridine triggered significant attraction ( Figure 1B ). By contrast, combinations of spermidine and pyridine did not elicit any attraction ( Figure 1B ). The attraction of the "Phe+Spe" blend was significantly higher than that of each of the "Phe+Spe+Pyr" and the "Spe+Pyr" combinations ( Figure 1B ). Likewise, the "Phe+Pyr" blend was more attractive than the "Spe+Pyr" combination. ( Figure 1B ).

Next, we tested the blend of all three odorants against each individual component. We found that the blend was less attractive than phenylacetaldehyde alone but attracted a similar number of flies as spermidine or pyridine alone ( Figure 1C ). This suggests that the addition of spermidine and pyridine to the blend may reduce its effectiveness.

To investigate this possibility, we conducted another set of experiments where mated females were offered a choice between two-odorant blends and their individual components. We found that blends of phenylacetaldehyde with either spermidine ( Figure 1D ) or pyridine ( Figure 1E ) were preferred over each of their respective individual components. However, the blend of spermidine and pyridine was not preferred over either odorant alone ( Figure 1F ).

These findings demonstrate that phenylacetaldehyde is the most effective attractant among tested odorants for mated females. The addition of spermidine or pyridine to phenylacetaldehyde enhances attraction, but these two compounds, when combined in blends, fail to elicit strong behavioral responses.

## Methods

**Drosophila stocks**

*D. suzukii* stock was collected in Connecticut in 2016 and has since then been reared on corn syrup and soy flour culture medium at 24 °C and 50% relative humidity in a 12:12 hr light-dark cycle. Flies aged 5-7 days were used in all experiments.

**Odorants**

Phenylacetaldehyde (Cat.# W287407), spermidine (Cat.# S2626), and pyridine (Cat.# 27040) were purchased at the highest available purity from MilliporeSigma.

**Two-choice trap assay**

The two-choice trap assay was described in Xue et al. (2024). Briefly, it consisted of a plastic pot with snap lid containing two trap cups. The traps are made from virgin polypropylene vials and white screw caps. The vials measure 4.3 cm in height and have a diameter of 3.5 cm. The screw caps are 1.1 cm in height with a diameter of 3.5 cm. Each cap has a 0.5 cm diameter hole through which a 1 ml filter tip entry extends into the trap. Twenty fed, mated female flies were introduced into the plastic pot, which was then closed with the lid and left for 24 hours in the dark. Flies enter traps via a 1 ml filter tip that is inserted through a hole in the middle of the trap cap. A preference index was calculated as (number of flies in the trap containing 1% agar mixed with 2% sucrose and an odorant blend−number of flies in the trap containing 1% agar mixed with 2% sucrose and the solvent control or a single odorant)/(total number of flies).

**Statistical analysis**

Statistical tests were performed in GraphPad Prism (version 10.0.1 (316)).
